# Supplementary material for: Nonlinear photovoltaic effects in monolayer semiconductor and layered magnetic material hetero-interface with P- and T-symmetry broken system
Source: Nat Commun. 2025 May 24;16:4827. doi: 10.1038/s41467-025-58918-9 (PMC12103524; doi:10.1038/s41467-025-58918-9)
Supplement: Supplementary file 1 — Supplementary Information [file 41467_2025_58918_MOESM1_ESM.pdf]

Supplementary Information for

Nonlinear photovoltaic effects in monolayer semiconductor and layered magnetic material hetero-interface with *P*- and *T*- symmetry broken system

Shuichi Asada[1], Keisuke Shinokita[1], Kenji Watanabe[2],

Takashi Taniguchi[3], and Kazunari Matsuda[1]

[1]*Institute of Advanced Energy, Kyoto University, Uji, Kyoto 611-0011, Japan*

[2]*Research Center for Functional Materials, National Institute for Materials Science, 1-1 Namiki, Tsukuba, Ibaraki 305-0044, Japan*

[3]*International Center for Materials Nanoarchitectonics, National Institute for Materials Science, 1-1 Namiki, Tsukuba, Ibaraki 305-0044, Japan*

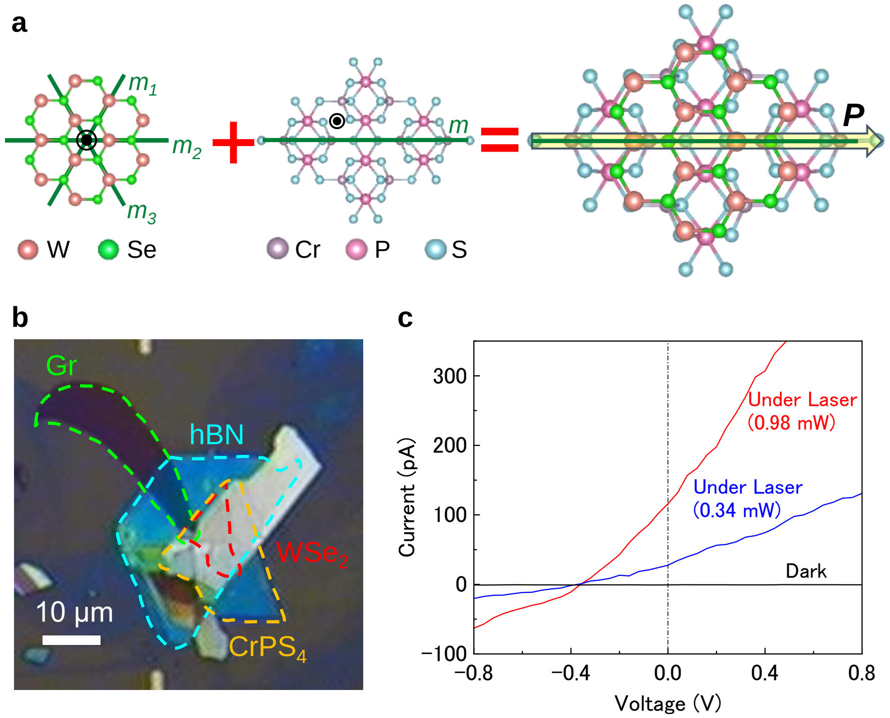

Fig. S1 **(a)** Schematic of crystal structure of 1L-WSe_2, $CrPS_4$ and WSe_2/$CrPS_4$ vdW heterostructure. The green lines and black circle dots show the axis of inversion symmetry and the center of rotational symmetry, respectively. In the vdW heterostructure, the inversion symmetry is reduced to left in only one-mirror plane and rotational symmetry is vanished. The induced polarization by the broken inversion symmetry is indicated by a yellow arrow. **(b)** Optical image of 1L-WSe_2/$CrPS_4$ vdW heterostructure. The monolayer WSe_2 and $CrPS_4$ are highlighted in red and orange dotted lines, respectively. Moreover, *h*-BN and graphene are highlighted in blue and green dotted lines, respectively. The scale bar of 10 μm is shown in the image. **(c)** Excitation power dependence of *I*-*V* characteristics of WSe_2/$CrPS_4$ vdW heterostructure under laser illumination of 532 nm. The *I*-*V* curves show finite photocurrents at zero-bias voltage depending on the excitation laser power.

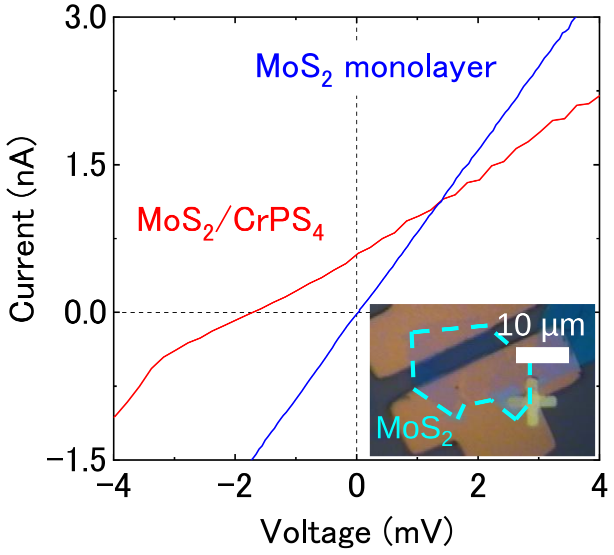

Fig. S2 *I*–*V* curves of the $MoS_2/CrPS_4$ heterostructure (red line) and monolayer MoS_2 device (blue line) under linearly polarized light (LPL) with a power of 600 μW. The inset shows a photograph of the monolayer MoS_2 device.

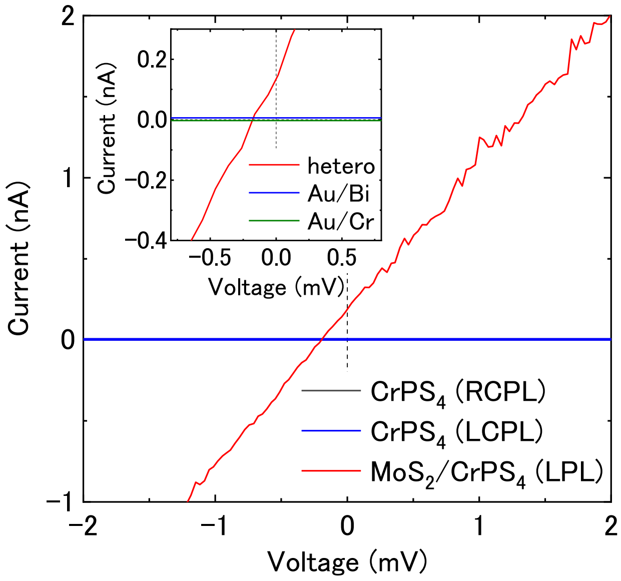

Fig. S3 *I*–*V* curves of bulk $CrPS_4$ under circularly polarized light (CPL) and the $MoS_2/CrPS_4$ heterostructure under LPL excitation conditions. The inset shows the *I*–*V* curves of the $MoS_2/CrPS_4$ heterostructure device and bulk $CrPS_4$ with different electrode materials.

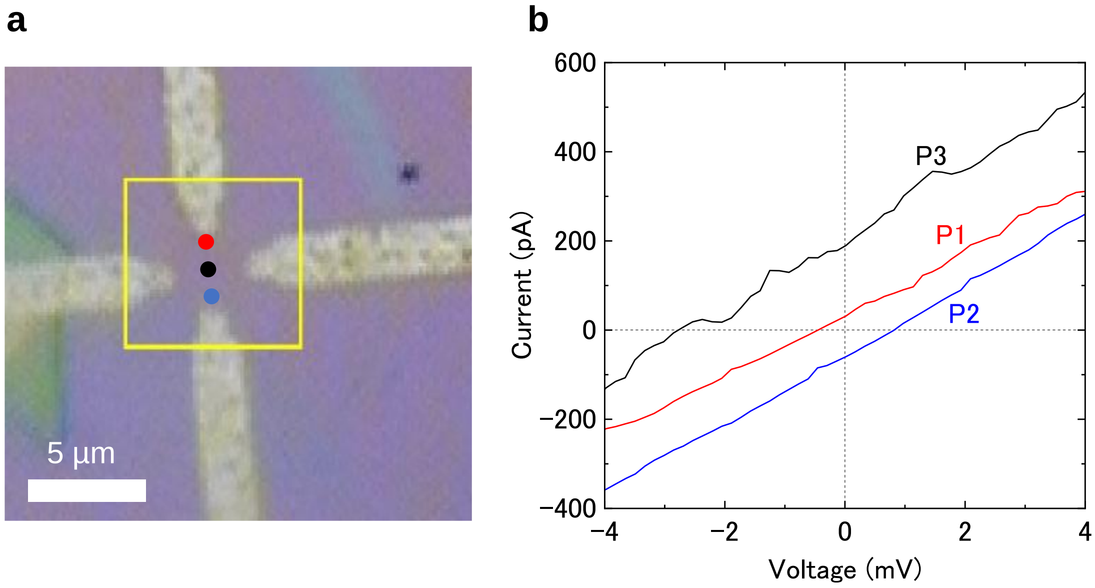

Fig. S4 (a) Optical image of 1L-MoS_2/$CrPS_4$ vdW heterostructure. The positions of photocurrent measurement are indicated by red, blue and black dot, respectively. The scale bar of 5 µm is shown in the image. **(b)** *I-V* characteristics of MoS_2/$CrPS_4$ vdW heterostructure measured at center of heterostructure and around electrodes. Each color of *I-V* curve corresponds to the measured position indicated in (a). Only P3 shows the large spontaneous photocurrent around the center of heterostructure region, and P1 and P2 show opposite spontaneous photocurrent around electrodes at zero-bias voltage from Schottky-barrier.

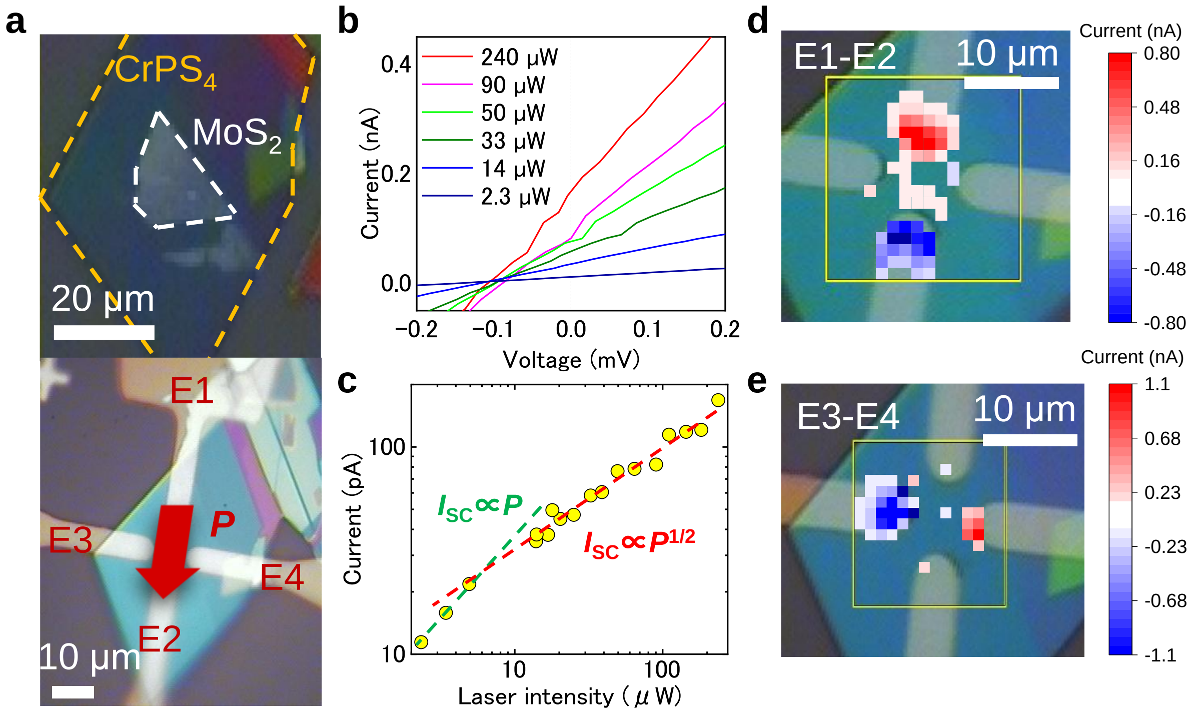

Fig. S5 (a) Optical image of different 1L-MoS_2/$CrPS_4$ vdW heterostructure. The MoS_2 monolayer and $CrPS_4$ are highlighted in white and orange dotted lines, respectively. Each electrode is assigned to E1–E4. The scale bar of 20 and 10 μm are shown in the images. **(b)** *I*-*V* characteristics of MoS_2/$CrPS_4$ vdW heterostructure under various excitation powers. **(c)** Excitation laser power dependence of spontaneous photocurrent. In the low power region below 10 μW, the photocurrent is proportional to the laser power, whereas the photocurrent shows the square-root power dependence above high power region above 10 μW. **(d)** and **(e)** Mapping of spontaneous photocurrent measured between E1 and E2 electrodes and E3 and E4 electrodes, respectively.

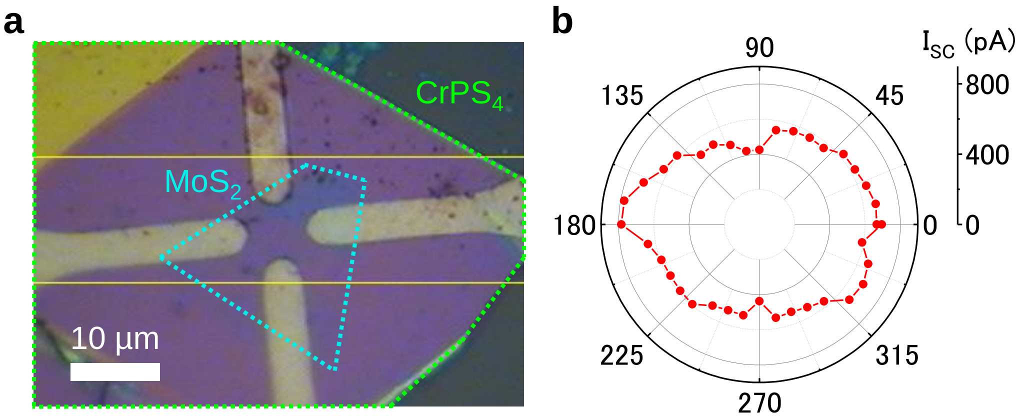

Fig. S6 (a) Optical image of another $MoS_2/CrPS_4$ heterostructure device. **(b)** Plot of the polarization-dependent spontaneous photocurrent.

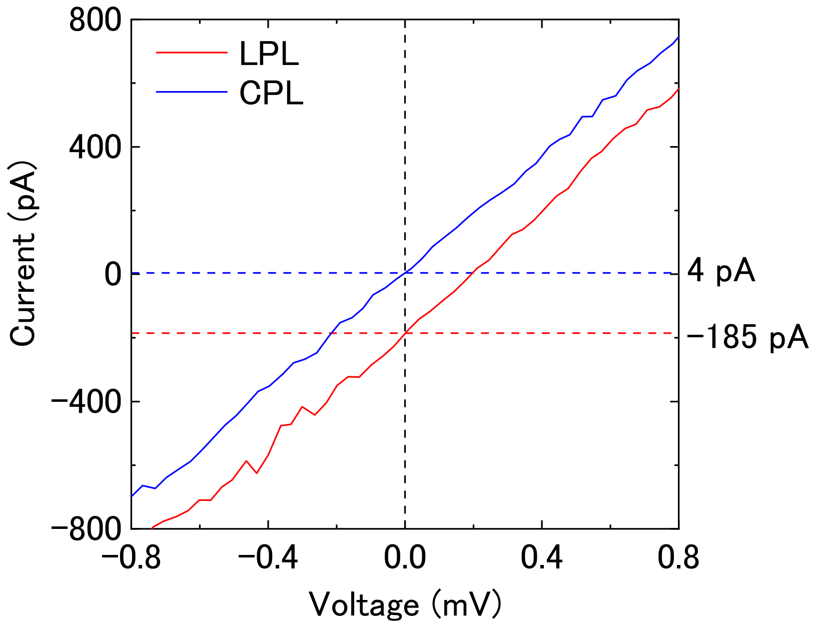

Fig. S7 *I*–*V* curves of the $MoS_2/CrPS_4$ heterostructure device with LPL (red line) and CPL (blue line).

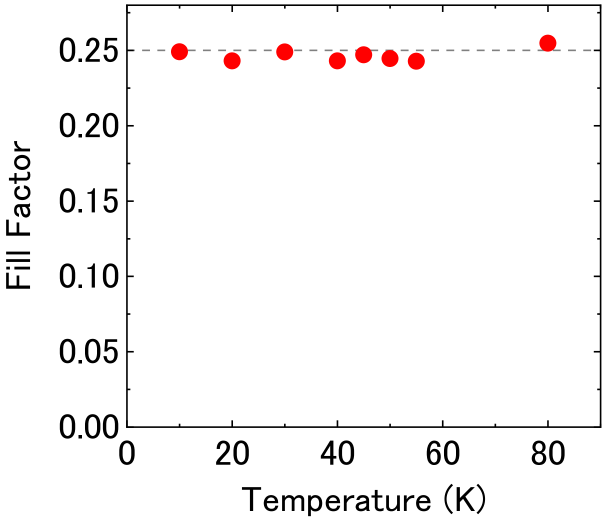

Fig. S8 Temperature dependence of the fill factor from the *I*–*V* curves of the $MoS_2/CrPS_4$ heterostructure device. The dotted line is a visual guide.

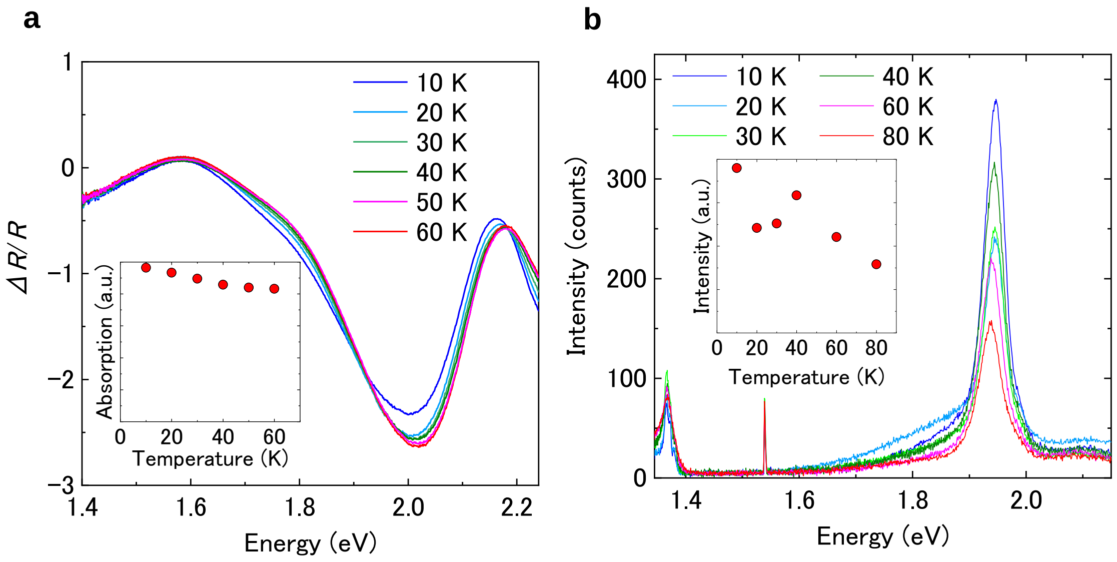

Fig. S9 (a) Temperature dependence of differential reflectance spectra in 1L-MoS_2/$CrPS_4$ vdW heterostructure. The differential reflectance is defined by $\Delta R/R = (R_{\mathrm{sub}} - R_{\mathrm{sample}})/R_{\mathrm{sub}}$, where the R_{sub} and R_{sample} correspond to the reflectance spectra of the substrate and sample, respectively. Inset shows the value of differential reflectance monitored at 2.3 eV. **(b)** Photoluminescence (PL) spectra of 1L-MoS_2/$CrPS_4$ vdW heterostructure. The inset shows the temperature dependence of PL intensity at 1.94 eV in monolayer MoS_2.

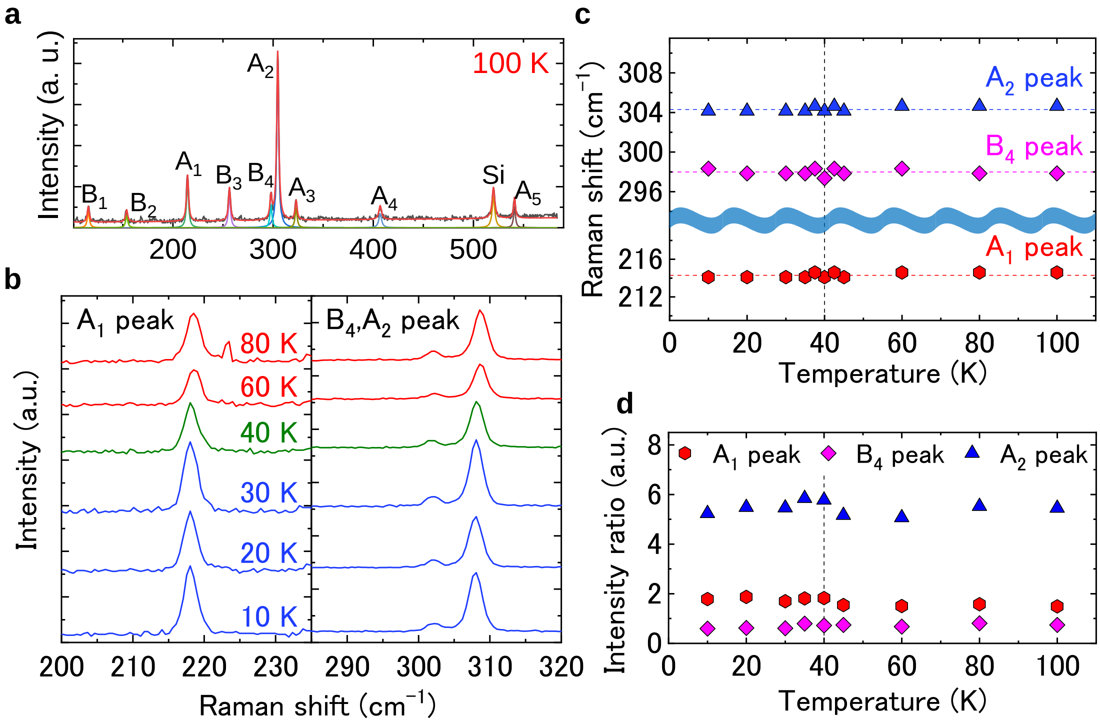

Fig. S10 (a) Raman scattering spectrum of 1L-MoS_2/$CrPS_4$ vdW heterostructure at 100 K. **(b)** Temperature dependence of Raman scattering spectra with A_1, B_4 and A_2 phonon peaks. **(c)** Temperature dependence of Raman peak position of A_2, B_4 and A_1 peak. **(d)** Temperature dependence of A_1, B_4 and A_2 Raman peak intensity normalized by Raman peak intensity of Si at 520 cm^{-1}.

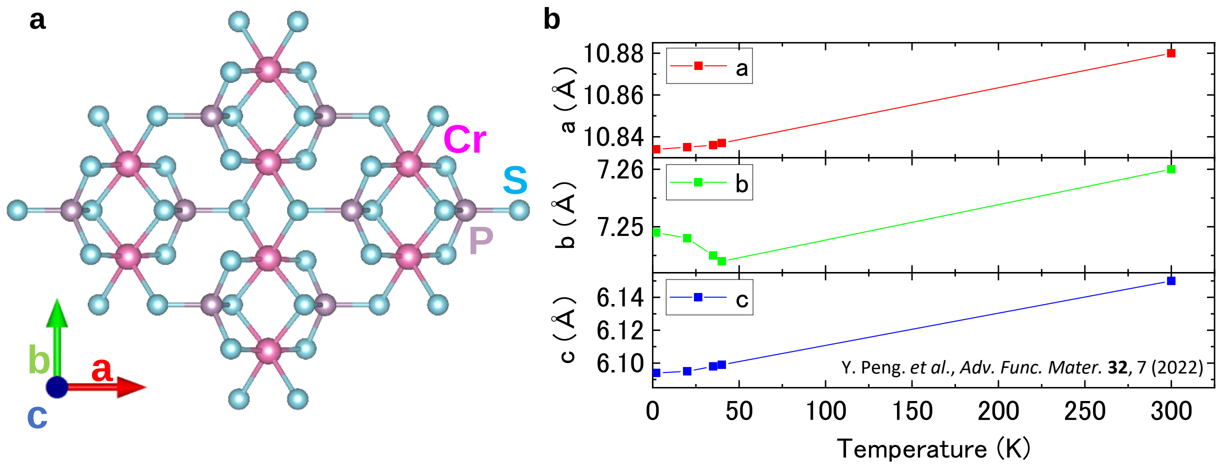

Fig. S11 (a) Schematic crystal structure of bulk $CrPS_4$ from top view. **(b)** Temperature dependence of lattice constants of bulk $CrPS_4$[1]. Spontaneous polarization at the interface of 1L-MoS_2/$CrPS_4$ heterostructure is expected along to *a*-axis of $CrPS_4$. The change of lattice constants of *a*-axis below Neel temperature of 40 K shows continuously changes from 10 to 300 K straddling 40 K (T_N).

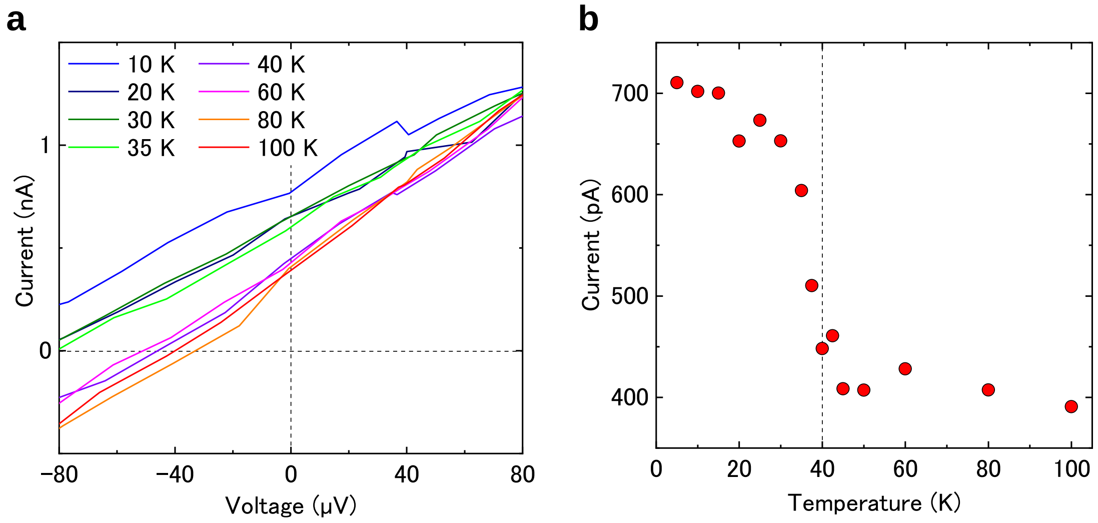

Fig. S12 (a) Temperature dependence of *I*-*V* characteristics of another 1L-MoS_2/$CrPS_4$ vdW heterostructure under the light illumination from 10 to 100 K. **(b)** Spontaneous photocurrent at zero-bias voltage as a function of temperature. The black dotted line at 40 K indicates Neel temperature of $CrPS_4$.

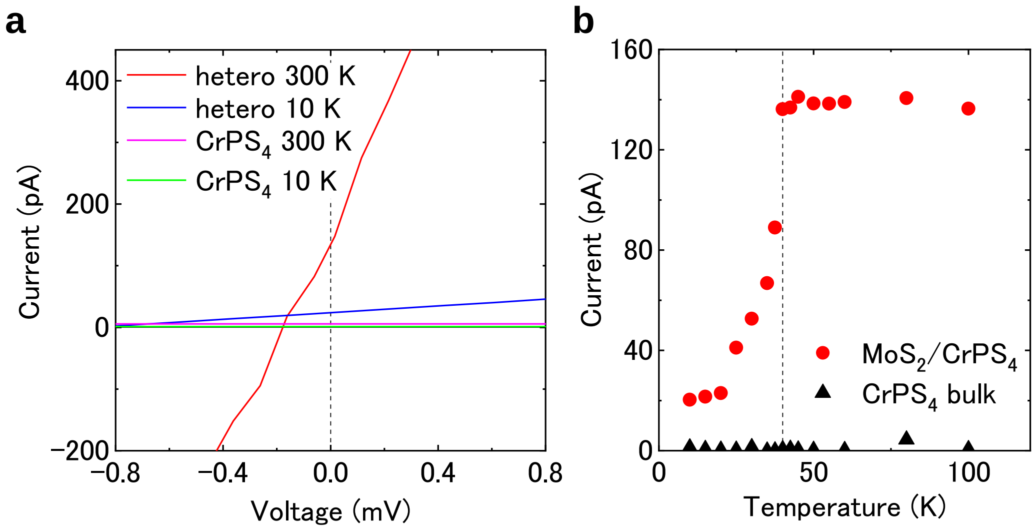

Fig. S13 (a) *I–V* curves of the bulk $CrPS_4$ and $MoS_2/CrPS_4$ heterostructures at 10 (below T_N) and 300 K (above T_N), excited by LPL. **(b)** Temperature dependence of the spontaneous photocurrent of the $MoS_2/CrPS_4$ heterostructure (red circles) and bulk $CrPS_4$ (black triangle)

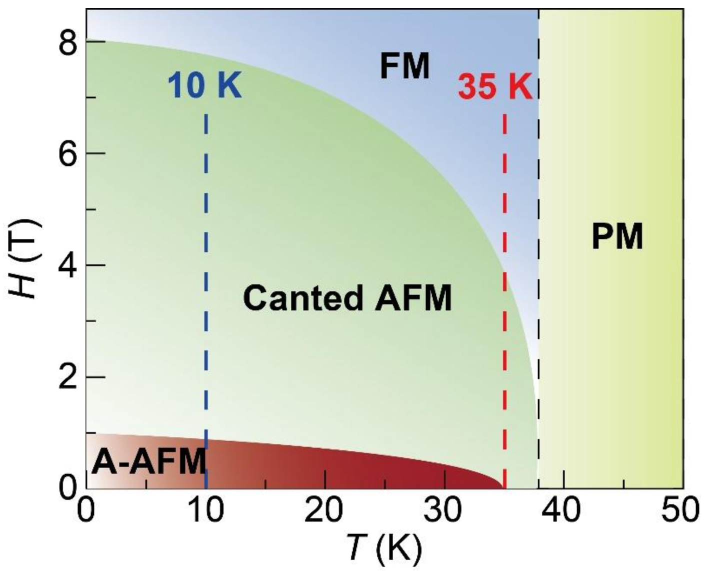

Fig. S14 Magnetic phase of $CrPS_4$ as a function of temperature and external magnetic field[2]. The experimental conditions at 10 and 35 K followed by the external magnetic field are shown by the dotted lines.

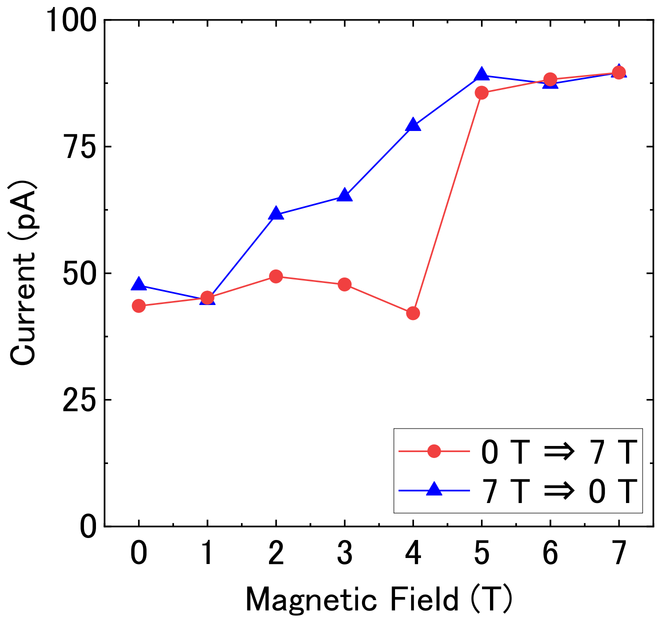

Fig. S15 Spontaneous photocurrent hysteresis in the $MoS_2/CrPS_4$ heterostructure when the external magnetic field was swept from 0 to 7 T and from 7 to 0 T.

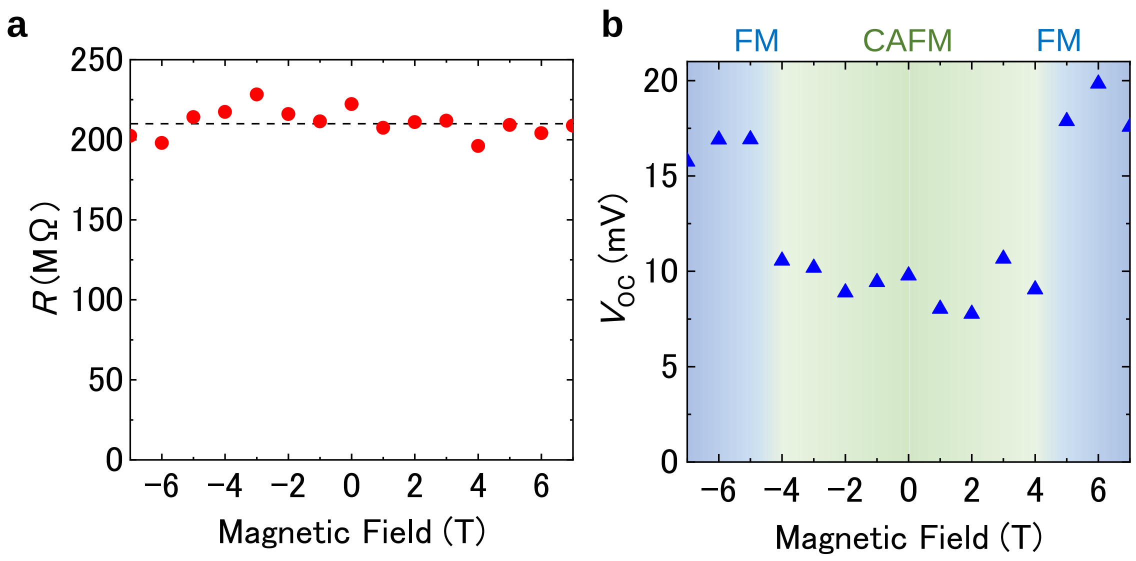

Fig. S16 (a) *R* and **(b)** V_{OC} as a function of the external magnetic field at 35 K.

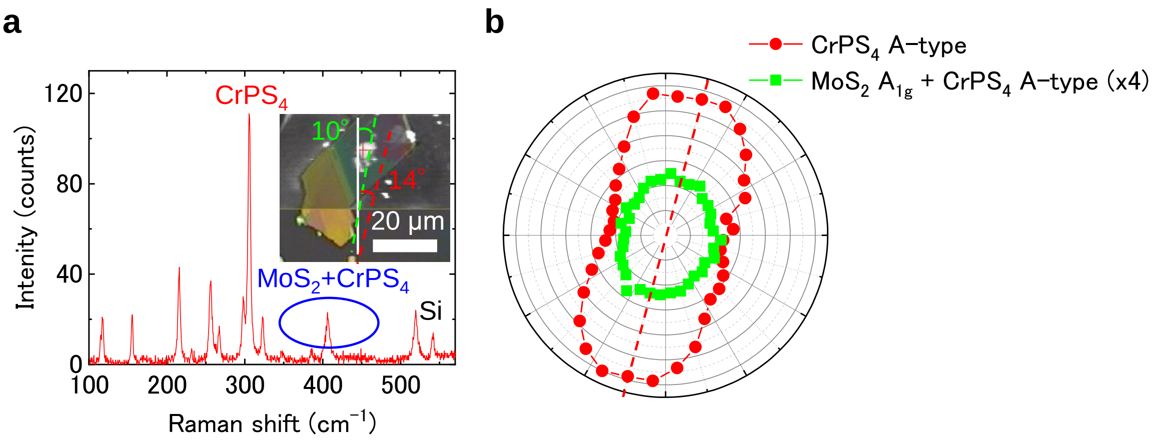

Fig. S17 (a) Raman scattering spectrum of the $MoS_2/CrPS_4$ heterostructure. The inset shows an optical image of the measured heterostructure. The relative crystal angles are indicated in the optical image. **(b)** Polar plot of the polarized Raman scattering intensity as a function of the angle of linearly polarized light: $CrPS_4$ A-type peak at 306 cm^{-1} (red), mixed peak of MoS_2 A_{1g} and $CrPS_4$ A-type peak at 406 cm^{-1} (green).

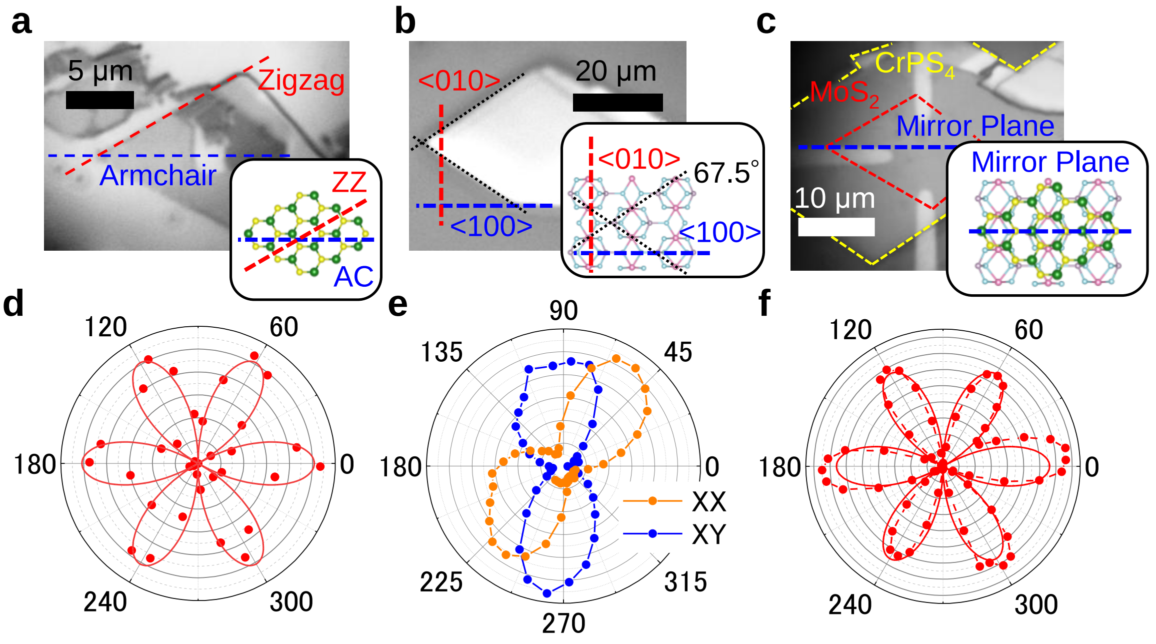

Fig. S18 Optical images of the **(a)** MoS_2 monolayer, **(b)** $CrPS_4$ bulk, and **(c)** $MoS_2/CrPS_4$ heterostructure samples. Polar plot of the SHG intensity of the **(d)** MoS_2 monolayer, **(e)** $CrPS_4$ bulk, and **(f)** $MoS_2/CrPS_4$ heterostructure. Each inset shows a schematic of the crystal structure and characteristic crystal axes, as shown by the dotted lines. The fitted linzAAZAZZes of the polar plots are shown, and the mirror plane angle is calculated from the fitting result.

	Shift current	Injection current	Observed
**Non magnetic**	○	×	Only shift current
**Magnetic**	○	○	Shift current<br>+<br>"Magnetic"<br>injection current

Table. R1 Relationship between the shift current, injection current and magnetic phase of $CrPS_4$.

REFERENCES

1. Y. Peng. *et al*., Controlling spin orientation and metamagnetic transitions in anisotropic van der Waals antiferromagnet $CrPS_4$ by hydrostatic pressure, *Adv. Func. Mater.* **32**, 7 (2022)

2. Y. Peng, *et al.,* Magnetic Structure and Metamagnetic Transitionsin the van der Waals Antiferromagnet $CrPS_4$, *Adv. Mater.* **32**, 28 (2020)
